# Development of the Intelligent Pneumatic Sewing Platform for Mask Production

**DOI:** 10.1109/ACCESS.2020.3013636

**Published:** 2020-08-03

**Authors:** Hao-Ting Lin

**Affiliations:** Department of Bio-Industrial Mechatronics EngineeringNational Chung Hsing University 34916 Taichung 402 Taiwan

**Keywords:** COVID-19, mask sewing production, pneumatic servo system, real-time control

## Abstract

This study fabricated a two-dimensional pneumatic servomechanism-based mask sewing platform. The platform has a simple structure, operates quickly, and is energy-efficient. SOLIDWORKS was used to design the platform, which comprises two rod-less pneumatic cylinders, one auxiliary cylinder, and a fabric-clamping mechanism. These parts are integrated with a sewing machine. The mask sewing platform has two degrees of freedom, as indicated by a mobility analysis of its mechanism. To control the highly nonlinear and time variant pneumatic system, a mathematical model for the pneumatic servo control system was developed and then analyzed in analytical form. An intelligent parameter adjustment feature with a sliding-mode controller was formulated to control the mask sewing platform. An experimental mask sewing platform was then designed and constructed. Finally, a personal computer–based system was developed using MATLAB Simulink for real-time control of the platform. The hybrid and fifth-order paths were successfully implemented in the test rig, and this study’s platform was experimentally verified as being capable of automatically producing masks.

## I. Introduction

Coronavirus disease 2019 (COVID-19) has become a global pandemic. The symptoms and severity of COVID-19 vary between individuals. It is currently believed that asymptomatic patients can also spread the disease, and symptoms of COVID-19 have been mild (mostly flu-like) in most instances [1], [2]. COVID-19 is primarily transmitted via droplets from a patient’s coughs or sneezes. Precautions must be taken to prevent the spread of COVID-19, and the wearing of masks in high-risk environments—such as in hospitals or public spaces—has been a key measure. This measure, however, has stressed the mask and textile industry, particularly with regard to labor requirements; this threatens to compromise production efficiency and quality, especially considering the increase in basic wages. This strain on labor must be addressed, and various studies have formulated methods that can do so. For example, Zoumponos and Aspragathos [3] proposed using fuzzy logic to program robots for use in sewing tables. Specifically, following parameter processing and data accumulation, fuzzy logic can then be used to develop data-rule algorithms, which overcome uncertainty in data collection. In Zoumponos and Aspragathos’s experiments, this fuzzy-logical method could be used to establish a complete path system, guiding robots to designated fabric locations (on fabrics of various sizes) to complete tasks. Panagiotis *et al.* [4] studied the application of stretchable materials to robots. They concluded that problems with stretchable materials involved their unpredictable nonlinearity, complex mechanical properties, and bending deformation. Fabric sewing is a delicate process because fabrics can change shape, and even twist, when forces (regardless of magnitude) are applied; this sensitivity to external forces makes automated sewing systems difficult to develop. However, automated sewing systems for manufacturing flexible-apparel products are essential because of rapid changes in consumer tastes, particularly in fashion and new fabric materials. Eric and Frank [5] investigated clothing production simulation systems. Specifically, they visually guided sewing robots along motion paths, which were defined using visual information and the coordinates of fabric edges. Subsequently, they conducted experiments involving shape analyses and motion control algorithms; their results indicated that visual guidance is the most effective method for guiding robot movements. Xiaoji and Kaiyong [6] argued that the stitching process is a crucial step that directly affects the shoemaking process, particularly the appearance of the finished shoe. Therefore, they introduced an automated shoe stitching process. Furthermore, in a previous work, the authors of the present study proposed a method where shoe upper images are input into an automatic seam processor to output digital control codes. The processor then uses a charge-coupled device camera to capture upper images before performing noise removal, binarization, edge detection, and edge thinning. Basic geometric sewing parameters, such as the upper contour, are captured from the upper images. Ryder *et al.* [7] controlled fabrics by replacing sewing machines with a servo-controlled robot gripper. They used a mechanical visual system to track the outlines of fabrics in order to identify detailed fabric locations and prevent fabric deformation. Their high-precision and high-acceleration location control system favorably controlled fabric location and tension.

Since the 1950s, robots performing tasks in the industry have been powered pneumatically. Because of improvements in computing, pneumatic systems and components have been more widely utilized in industry and in automation technology. Furthermore, control theories and methods with regard to automation have allowed pneumatic systems to behave with greater linearity and predictability. Consequently, the use of pneumatic actuators and valves, particularly in automated, robotic sewing production, has various advantages—such as greater responsiveness, safety, stability, energy efficiency, and ease of maintenance. However, compressible fluid, pneumatic systems have the key disadvantage of being less accurate relative to electric motors. High nonlinearity, low stiffness, and low compressibility result in complexity when controlling a pneumatic system. Nonetheless, research on pneumatically driven robots in the automation industry remains fairly prolific despite these drawbacks.

Sliding-mode control is an effective control method that is adopted to handle the nonlinear behavior, model uncertainty, and bounded disturbances in a pneumatic system [8], [9]. In real-time control experiments, Lin [10] proposed a novel real-time path servo control system for a large-stroke asymmetric rod-less pneumatic system under variable loads in a hardware-in-the-loop simulation. Their results indicated that trajectory objectives in various strokes could be successfully implemented in their test rig. Saravanakumar *et al.* [11] reviewed recent research trends in servo pneumatic positioning systems; they focused on methods for diminishing nonlinearity in the pneumatic system to make the pneumatic servo system more efficient and accurate. Ren *et al.* [12]–[14] proposed adaptive backstepping control, fractional order sliding-mode control, and fractional order PID control for a pneumatic position servo system, and they implemented this approach in a test rig. Mu *et al.* [15] proposed and experimentally verified an intelligent position control mechanism, which was based on predictive fuzzy control, for a pneumatic servo system. Zhao *et al.* [16] investigated the positioning control of a rod-less cylinder in a pneumatic servo system with actuator saturation. In their experimental results, the system’s accuracy was 0.005 mm for a 200 mm step signal. Saravanakumar *et al.* [17] designed interconnected pneumatic cylinders to improve a pneumatic system’s performance with respect to rise time and overshoot.

Considering these advances in the literature, this study designed a novel two-dimensional pneumatic servome- chanism-based mask sewing platform (TDPSMSP). Mask manufacturers can incorporate this system into their automatic sewing and stitching machines to substantially reduce labor and time costs during mask fabric manufacturing; the TDPSMSP also facilitates the full automation of factory operations. Air is used to generate pressure energy to power the TDPSMSP because of its accessibility, safety, and compressibility. Consequently, the TDPSMSP has a simple and highly responsive structure, in addition to being clean, safe, cost-efficient, and energy-efficient. As for the TDPSMSP’s mechanical design, a rod-less pneumatic cylinder was selected as the actuator, and the advantages of the cylindrical shape (specifically, ease of assembly and simplicity in design) were used to provide a larger work space. The TDPSMSP’s mobility was analyzed using Kutzbach-Gruebler’s equation. Subsequently, a specimen machine was designed using SOLIDWORKS. To control the highly nonlinear and time-varying pneumatic system, a mathematical model for controlling the pneumatic servo system was developed and then analyzed. An intelligent parameter adjustment feature with a sliding-mode control was formulated to control the mask sewing platform. Next, a personal computer (PC)-based system-controlled TDPSMSP was developed using MATLAB Simulink. The experimental results indicated that masks are produced by the TDPSMSP through path generation and fifth-order polynomial trajectories for tracking and controlling with references.

## II. Structural Design of the TDPSMSP

**FIGURE 1.**
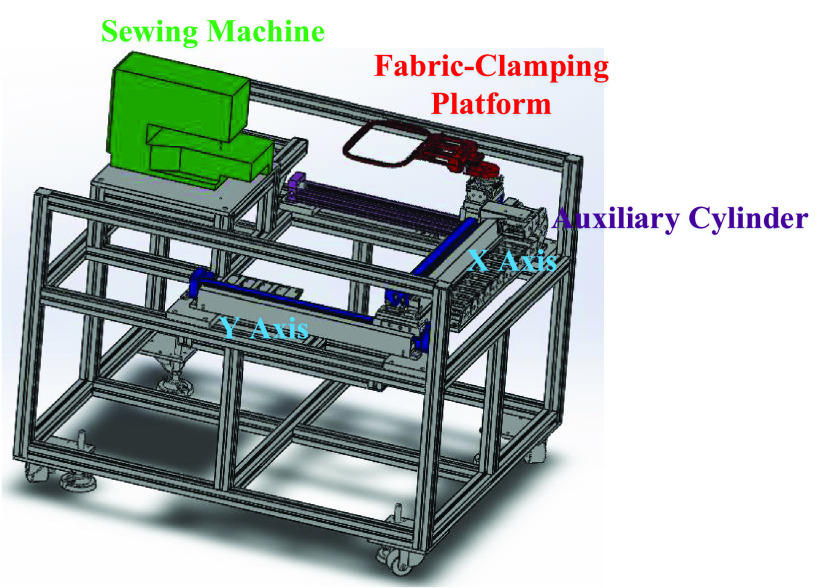
Two-dimensional pneumatic servomechanism-based mask sewing platform.

Figure 1 illustrates the structural design of the TDPSMSP, which mainly comprises two rod-less pneumatic cylinders, an auxiliary cylinder, a cloth-clamping platform, and a sewing machine. The proposed TDPSMSP is basically composed of two identical limbs and a moving platform. Two sliders are translated along the linear guide-ways by two one degree of freedom (DOF) prismatic joints driven by the pneumatic rod-less cylinders, input links. The part marked in green is the sewing machine, that marked in red is the fabric-clamping platform, that marked in blue is the rod-less pneumatic cylinder, and that marked in purple is the auxiliary cylinder. Sliders on the cylinders could move to control fabrics fed to the fabric-clamping platform, completing the sewing process.

**FIGURE 2.**
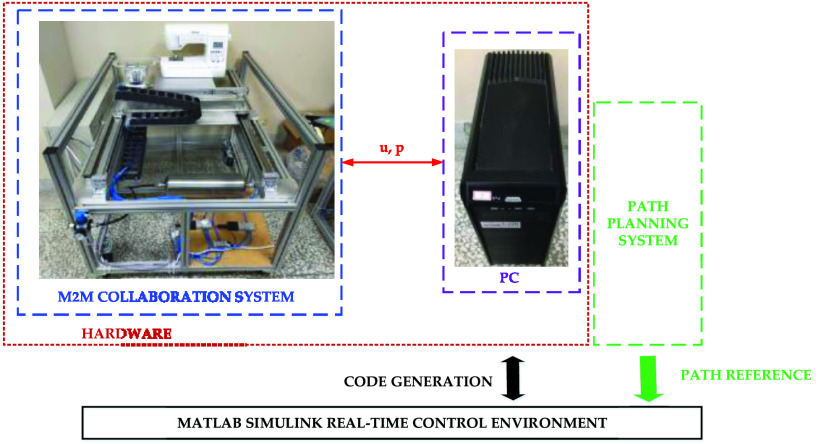
The layout of the pneumatic real-time servo control platform and the sewing machine.

Figure 2 is the layout of the pneumatic real-time servo control platform and the sewing machine. The M2M collaboration system is the pneumatic real-time servo control platform with the sewing machine. The rod-less pneumatic cylinder and auxiliary cylinder, model numbers DGC-25-500-GF-PPV-A and DGC-25-500-FA-P, respectively, were manufactured by FESTO. In this experiment, the machine pressure source was set at 6 bar. Furthermore, this study used pneumatic proportional servo valves (model: FESTO MPYE-5-1/8-HF-010-B) to control the pneumatic cylinders. The input and median voltage were set at 0–10 V and 5 V, respectively. An AD/DA interface card (PCI-1723) manufactured by Advantech was employed for signal control, whereas a counting card, manufactured by NI (NI-6601), was utilized for receiving optical ruler (JENA LIA 20) signals. A PC-based system was developed using MATLAB Simulink, in which the main design flow entailed using MATLAB’s.M files to set parameters and design paths and using a program created using Simulink to obtain.M file parameters for subsequent calculations. Control signals were transmitted to the AD/DA card to control the pneumatic proportional servo valve output flows and directions, thereby enabling control of the air pressure rod-less pneumatic cylinder flows and directions. The optical ruler was then used to read the displacement of the rod-less pneumatic cylinder and issue immediate feedback. The sampling frequency was set at 1 kHz. A sewing machine was manufactured by Brother, model numbers Innovis F410. The preset and actual trajectories were then compared, and discrepancies were returned to the controller to control subsequent operation. By constantly correcting and compensating values, errors were corrected to approach target values. In this study, TDPSMSP experiments were performed using real-time trajectory-tracking control to prevent data (read using the optical ruler) losses when movement speed was excessively rapid. Table 1 shows specifications of the M2M collaboration system via the pneumatic real-time servo control platform and the sewing machine. When sewing the mask, the tooth of the sewing machine will face downward. Thus, the feed speed of fabric will be unaffected by the sewing machine. In this study’s mask product experiment, the feed speed of fabric could reach 26 mm/s, which was greater than the 14.2 mm/s sewing speed of this sewing machine.

**TABLE 1:** Specifications of the M2M Collaboration System Via the Pneumatic Real-Time Servo Control Platform and the Sewing Machine

Component	Specification
Rod-less pneumatic cylinder	Piston diameter: 25 mm
	Stroke: 500 mm
Auxiliary cylinder	Stroke: 500 mm
Sewing Machine	Sewing speed: 70 – 850 stitches per minute
	Maximum stitch width: 7 mm
Pneumatic proportional directional control valve	Valve function: 5/3 way
	Input voltage: 0 – 10 V
AD/DA card	16-bit, 8-ch analog output PCI card with 16-ch digital I/O
Counter card	4-ch 32-bit counter with 20 MHz maximum source frequency
Optical encoder	Range: 500 mm
	Resolution: $1~\mu \text{m}$

## III. Analysis and Control of the Pneumatic System Mathematical Model

### A. Rod-Less Pneumatic System Mathematical Model and Control

To yield accurate mathematic models, this study firstly investigate rod-less pneumatic system mathematical models. The dynamics of a rod-less pneumatic system can be mainly divided into two parts: the pneumatic servo valve and the pneumatic cylinder. In the pneumatic servo valve, the opening area of the pneumatic valve orifice is controlled by input command signal. And air flow is affected through the pneumatic servo valve. As air flows into and out of the cylinder, pressure differences between pneumatic cylinder chambers will cause motions of the cylinder piston. We assume that the valve’s dynamic behavior can be expressed using a zero order model as

\begin{equation*} A_{o}\left ({t }\right)=\mathrm {K}u\left ({t }\right)\tag{1}\end{equation*}

where $A_{o}\left ({t }\right)$ is the open area of the pneumatic valve, K represents the open area-voltage gain, and $u\left ({t }\right)$ is the control input of a pneumatic servo valve. Mass flow rate equations for pneumatic cylinder chambers can be written as follows

\begin{align*} \dot {\boldsymbol {m}}\left ({\boldsymbol {t} }\right)=&\frac {\boldsymbol {C}_{\boldsymbol {d}}\boldsymbol {C}_{\mathbf {0}}\boldsymbol {A}_{\mathbf {0}}\boldsymbol {(t)}\boldsymbol {P}_{\boldsymbol {u}}\boldsymbol {(t)}\tilde {f} \left(\frac {\boldsymbol {P}_{\boldsymbol {d}}\left ({\boldsymbol {t} }\right)}{\boldsymbol {P}_{\boldsymbol {u}}\left ({\boldsymbol {t} }\right)}\right)}{\sqrt {\boldsymbol {T}_{\boldsymbol {u}}}} \tag{2}\\&\hspace {-3pc}\hat {f}\left ({P_{a}\left ({t }\right),P_{s}\left ({t }\right),P_{e}\left ({t }\right) }\right) \\=&\left \{{ {\begin{array}{cccccccccccccccccccc} \displaystyle \frac {P_{s}\left ({t }\right)\tilde {f}\left ({\frac {P_{a}\left ({t }\right)}{P_{s}\left ({t }\right)} }\right)}{\sqrt {T}_{s}}&\mathrm {A~is~a~driving~chamber}\\ \displaystyle \frac {P_{a}\left ({t }\right)\tilde {f}\left ({\frac {P_{e}\left ({t }\right)}{P_{a}\left ({t }\right)} }\right)}{\sqrt {T}_{a}}&\mathrm {B~is~a~driving~chamber}\\ \end{array}} }\right \}\quad \tag{3}\\&\hspace {-3pc}\hat {f}\left ({P_{b}\left ({t }\right),P_{s}\left ({t }\right),P_{e}\left ({t }\right) }\right) \\=&\left \{{ {\begin{array}{cccccccccccccccccccc} \displaystyle \frac {P_{s}\left ({t }\right)\tilde {f}\left ({\frac {P_{b}\left ({t }\right)}{P_{s}\left ({t }\right)} }\right)}{\sqrt {T}_{s}}&\mathrm {B~is~a~driving~chamber}\\ \displaystyle \frac {P_{b}\left ({t }\right)\tilde {f}\left ({\frac {P_{e}\left ({t }\right)}{P_{b}\left ({t }\right)} }\right)}{\sqrt {T}_{b}}&\mathrm {A~is~a~driving~chamber}\\ \end{array}} }\right \}\tag{4}\end{align*}

where $\dot {\boldsymbol {m}}\left ({\boldsymbol {t} }\right)$ is mass flow rate, $\boldsymbol {C}_{\boldsymbol {d}}= 0.8$ is the displacement coefficient, $\boldsymbol {C}_{\mathbf {0}}$ is the flow rate parameter, $\boldsymbol {P}_{\boldsymbol {u}}\left ({\boldsymbol {t} }\right)$ is up-stream pressure, $\boldsymbol {P}_{\boldsymbol {d}}\left ({\boldsymbol {t} }\right)$ is down-stream pressure, $\boldsymbol {P}_{\boldsymbol {S}}\left ({\boldsymbol {t} }\right)=\mathbf {6}\times {\mathbf {10}}^{\mathbf {5}}$ Pa is supplied pressure, $\boldsymbol {P}_{\boldsymbol {e}}\left ({\boldsymbol {t} }\right)=\mathbf {1}\times {\mathbf {10}}^{\mathbf {5}}$ Pa is external pressure, $\boldsymbol {P}_{\boldsymbol {a}}\left ({\boldsymbol {t} }\right)$ and $\boldsymbol {P}_{\boldsymbol {b}}\left ({\boldsymbol {t} }\right)$ are chamber pressures. Assuming no temperature dependency, $\boldsymbol {T}_{\boldsymbol {s}}=\boldsymbol {T}_{\boldsymbol {a}}=\boldsymbol {T}_{\boldsymbol {b}}=\mathbf {293 K}$ are supplied air temperature and temperatures of chambers A and B.

Assuming an adiabatic process, fluid continuity equations are shown as

\begin{align*} \dot {m_{a}}\left ({t }\right)=&\frac {{\dot {P_{a}}\left ({t }\right)V}_{a}(t)}{kRT_{s}}+\frac {{\dot {V_{a}}\left ({t }\right)P}_{a}(t)}{RT_{s}} \tag{5}\\ \dot {m_{b}}\left ({t }\right)=&\frac {{\dot {P_{b}}\left ({t }\right)V}_{b}(t)}{kRT_{s}}+\frac {{\dot {V_{b}}\left ({t }\right)P}_{b}(t)}{RT_{s}}\tag{6}\end{align*}

where $\dot {m_{a}}(t)$ and $\dot {m_{b}}(t)$ are mass flow rate of chambers A and B, $k=1.4$ is the thermal constant, $\mathrm {R}= 287$ J/(kg$\cdot \text{K}$) is the ideal gas constant, $V_{a}(t)$ and $V_{b}(t)$ are chamber volumes A and B. Applying Newton’s second law of motion to rod-less pneumatic cylinder’s mass, we get

\begin{align*}&\left ({\mathrm {A}P_{a}\left ({t }\right)-\mathrm {A}P_{b}\left ({t }\right) }\right)sgn\left ({x\left ({t }\right) }\right)-K_{f}\dot {x}\left ({t }\right) \\&\quad -\,K_{c}\left ({x\left ({t }\right) }\right)S\left ({\dot {x}\left ({t }\right),P_{a}\left ({t }\right),P_{b}\left ({t }\right) }\right)-Mg=M\ddot {x}(t)\tag{7}\end{align*}

where $K_{f}$ is the coefficient of viscosity, and $K_{c}$ is the Coulomb’s friction coefficient. $M$ is the mass of the rod-less pneumatic cylinder piston (kg).

From Eq. (1)–Eq. (7), the mathematical model of the rod-less pneumatic system can be expressed as a fourth-order model basically including the pneumatic servo valve system and the pneumatic actuator system. Its state equation is expressed as follows:

\begin{align*}&\hspace {-1pc}\left [{ {\begin{array}{l} {\begin{array}{cccccccccccccccccccc} \dot {x_{1}}(t)\\ \dot {x_{2}}(t)\\ \dot {x_{3}}(t)\\ \end{array}} \\ \dot {x_{4}}(t) \\ \end{array}} }\right] \\=&\left [{\!\! {\begin{array}{l} {\begin{array}{cccccccccccccccccccc} x_{2}(t)\\ \frac {\left ({Ax_{3}\left ({t }\right)-Ax_{4}\left ({t }\right) }\right)sgn\left ({x_{1}\left ({t }\right) }\right)-K_{f}x_{2}\left ({t }\right)-K_{c}\left ({x_{1}\left ({t }\right) }\right)S\left ({x_{2}\left ({t }\right),x_{3}\left ({t }\right),x_{4}\left ({t }\right) }\right)}{M}\\ \frac {-kx_{2}(t)x_{3}(t)}{x_{1}\left ({t }\right)+\Delta }+\frac {kRTC_{d}C_{0}wu(t)\hat {f}(x_{3}\left ({t }\right),P_{S}\left ({t }\right),P_{e}\left ({t }\right))}{A(x_{1}\left ({t }\right)+\Delta)}\\ \end{array}} \\ \frac {kx_{2}(t)x_{4}(t)}{l-x_{1}\left ({t }\right)-\Delta }+\frac {kRTC_{d}C_{0}wu(t)\hat {f}(x_{4}\left ({t }\right),P_{S}\left ({t }\right),P_{e}\left ({t }\right))}{A(l-x_{1}\left ({t }\right)-\Delta)} \\ \end{array}}\!\! }\right]\!\!\!\! \\\tag{8}\end{align*}

$\Delta $ is an equivalent extra length to the cylinder, $l$ is the total travel distance of the rod-less pneumatic cylinder. Additionally, $x_{1}$ is the amount of rod-less pneumatic cylinder piston movement, $x_{2}$ is the rod-less pneumatic cylinder piston movement speed, $x_{3}$ is the pressure of rod-less pneumatic cylinder Chamber A, $x_{4}$ is the pressure of rod-less pneumatic cylinder Chamber B.

To allow the pneumatic real-time servo control platform to control locations and trajectories, $u(t)$ is a control input adopted an intelligent parameter adjustment with sliding mode control to solve problems of nonlinearity, time variant, and external interferences used in pneumatic servo systems. From Eq. (8), the system can also be expressed as

\begin{align*} \dot {\boldsymbol {x}}\left ({t }\right)=&f\left ({\boldsymbol {x},t }\right)+g\left ({\boldsymbol {x},t }\right)u\left ({t }\right) \\ y\left ({t }\right)=&h(\boldsymbol {x},t)\tag{9}\end{align*}

where

\begin{align*} f\left ({\boldsymbol {x},t }\right)=&\left [{ {\begin{array}{l} {\begin{array}{cccccccccccccccccccc} x_{2}(t)\\ \frac {{\begin{array}{l} \left ({Ax_{3}\left ({t }\right)-Ax_{4}\left ({t }\right) }\right)sgn\left ({x_{1}\left ({t }\right) }\right)-K_{f}x_{2}\left ({t }\right) \\ -K_{c}\left ({x_{1}\left ({t }\right) }\right)S\left ({x_{2}\left ({t }\right),x_{3}\left ({t }\right),x_{4}\left ({t }\right) }\right) \\ -M_{L} \\ \end{array}}}{M}\\ \frac {-kx_{2}(t)x_{3}(t)}{x_{1}\left ({t }\right)+\Delta }\\ \end{array}} \\ \frac {kx_{2}(t)x_{4}(t)}{l-x_{1}\left ({t }\right)-\Delta } \\ \end{array}} }\right] \\[8pt] g\left ({\boldsymbol {x},t }\right)=&\left [{ {\begin{array}{l} {\begin{array}{cccccccccccccccccccc} 0\\ 0\\ \frac {kRTC_{d}C_{0}w\hat {f}(x_{3}\left ({t }\right),P_{S}\left ({t }\right),P_{e}\left ({t }\right))}{A(x_{1}\left ({t }\right)+\Delta)}\\ \end{array}} \\ \frac {kRTC_{d}C_{0}w\hat {f}(x_{4}\left ({t }\right),P_{S}\left ({t }\right),P_{e}\left ({t }\right))}{A(l-x_{1}\left ({t }\right)-\Delta)} \\ \end{array}} }\right]\\[8pt] y\left ({t }\right)=&x_{1}(t)\end{align*}

The nonlinear time-varying terms $f\left ({\boldsymbol {x},t }\right)$ and $g\left ({\boldsymbol {x},t }\right)$ can be defined as unknown functions by the functional approximation technique with Fourier series. The response error can be written as

\begin{equation*} e\left ({t }\right)=y_{m}\left ({t }\right)-y\left ({t }\right)\tag{10}\end{equation*}

Define a second-order sliding surface as

\begin{equation*} s=a_{1}e\left ({t }\right)+a_{2}\dot {e}\left ({t }\right)+\ddot {e}\left ({t }\right)\tag{11}\end{equation*}

The control input is chosen as (12), shown at the bottom of the next page,

**Figure:** \begin{equation*} u\left ({t }\right)=\frac {-\widehat {\boldsymbol {W}_{F}^{T}}\boldsymbol {q}_{F}\left ({t }\right)-\sum \nolimits _{n=1}^{2} {a_{n}e^{\left ({n }\right)}-p_{21}e-p_{22}\dot {e}+y_{m}^{\left ({3 }\right)}\left ({t }\right)} -\frac {s}{2\rho ^{2}}}{\widehat {\boldsymbol {W}_{g}^{T}}\boldsymbol {q}_{g}(t)}\tag{12}\end{equation*}

where $\widehat {\boldsymbol {W}_{F}^{T}}$ and $\widehat {\boldsymbol {W}_{g}^{T}}$ are the estimates of $\boldsymbol {W}_{\boldsymbol {F}}^{\boldsymbol {T}}$and $\boldsymbol {W}_{\boldsymbol {g}}^{\boldsymbol {T}}$ respectively; $\boldsymbol {q}_{\boldsymbol {F}}\left ({\boldsymbol {t} }\right)$ and $\boldsymbol {q}_{\boldsymbol {g}}\boldsymbol {(t)}$ represents the orthogonal functions; $e\left ({t }\right)$ is the output error; $s$ is the sliding surface; $\rho $ is the natural number; $y_{m}\mathrm {(t)}$ is a given bounded reference objective; and $p_{2i}$ is the element.

The Lyapunov function candidate can be defined as follows:

\begin{equation*} \boldsymbol {V}=\frac {\mathbf {1}}{\mathbf {2}} \left(s^{2}+e^{T}Pe+\mathrm { }\widetilde {W_{F}^{T}}\gamma _{1}^{-1}\widetilde {W_{F}}+\widetilde {W_{g}^{T}}\gamma _{2}^{-1}\widetilde {W_{g}}\right)\tag{13}\end{equation*}

where $\widetilde {W_{F}}=\widehat {W_{F}}-W_{F}$ and $\widetilde {W_{g}}=\widehat {W_{g}}-W_{g}$. $P>0$ is a matrix of $p_{2i}$. The following intelligent parameter adjustment method is adopted:

\begin{align*} \dot {\widehat {W_{F}}}=&\boldsymbol {\gamma }_{\mathbf {1}}s\boldsymbol {q}_{F}\left ({t }\right) \tag{14}\\ \dot {\widehat {W_{g}}}=&\boldsymbol {\gamma }_{\mathbf {2}}s\boldsymbol {q}_{g}\left ({t }\right)\tag{15}\end{align*}

where $\boldsymbol {\gamma }_{\mathbf {1}}>0$ and $\boldsymbol {\gamma }_{\mathbf {2}}>0$ are the adjustment gain matrices.

The derivative of $\boldsymbol {V}$ is given as

\begin{align*} \dot {\boldsymbol {V}}=s\dot {s}+\frac {1}{2}\dot {e^{T}}Pe+\frac {1}{2}e^{T}P\dot {e}\mathrm { }+\widetilde {W_{F}^{T}}\gamma _{1}^{-1}\dot {\widetilde {W_{F}}}+\widetilde {W_{g}^{T}}\gamma _{2}^{-1}\dot {\widetilde {W_{g}}}\big) \\\tag{16}\end{align*}

Substituting (12), (14) and (15) into (16), we obtain:

\begin{align*} \dot {\boldsymbol {V}}=&-\frac {1}{2}\left({e^{T}\boldsymbol {Q}e+\left ({\frac {s}{\rho }-\rho w_{t} }\right)^{2}-\rho ^{2}w_{t}^{2}}\right) \\\le&-\frac {1}{2}(e^{T}\boldsymbol {Q}e-\rho ^{2}w_{t}^{2})\tag{17}\end{align*}

where $\boldsymbol {Q>0}$ satisfied $\boldsymbol {A}_{\mathbf {1}}^{\boldsymbol {T}}\boldsymbol {P}+\boldsymbol {P}\boldsymbol {A}_{\mathbf {1}}=-\boldsymbol {Q}$

Integrating (17), we obtain

**FIGURE 3.**
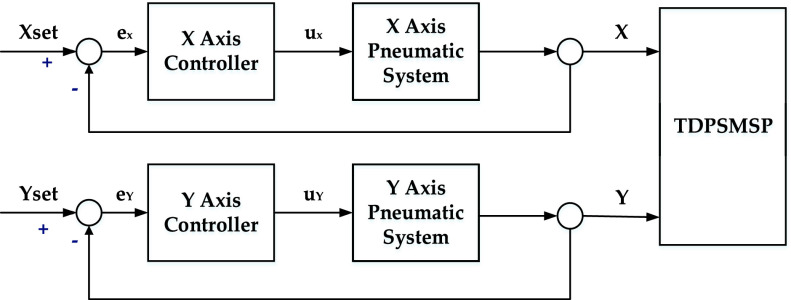
Two-dimensional pneumatic servomechanism-based mask sewing platform control system.

\begin{equation*} V(t)\le V(0)-\frac {1}{2}\int _{0}^{T} {(e^{T}\boldsymbol {Q}e-\rho ^{2}w_{t}^{2}})d\tau\tag{18}\end{equation*}

where $w_{t}$ is the actual upper bound for the approximation errors in practice. Figure 3 is a block diagram of the TDPSMSP control system, in which individual controls were used. Table 2 details the control parameters of the experiments. Also, $\begin{aligned} \boldsymbol {Q}=\left [{ {\begin{array}{cc} 1 & 2\\ 2 & 5\\ \end{array}} }\right] \end{aligned}$.

**TABLE 2:** Control Parameters of Experiments

	X axis	Y axis
$a_{1}$	500	500
$a_{2}$	50	50
$\rho $	$\frac {1}{\sqrt {500}}$	$\frac {1}{\sqrt {500}}$
$\gamma _{1}$	$100\times I_{23\times 23}$	$100\times I_{23\times 23}$
$\gamma _{2}$	$1.25\times {10}^{-4}{\times I}_{23\times 23}$	$1.25\times {10}^{-4}{\times I}_{23\times 23}$
$W_{F}$	0.01	0.01
$W_{g}$	8000	8000

### B. Degree of Freedom Analysis

This study used two rod-less pneumatic cylinders as the movement-driving units. The $x$-axis was loaded on the $y$-axis, and the two axes moved to feed fabric into the sewing machine. According to the definition of degrees of freedom for sewing machines, the TDPSMSP in this study was defined as a two-dimensional system, and its analysis was expressed as follows:

\begin{equation*} \mathrm {DOF}=\lambda _{F}\left ({n_{L}-1 }\right)-\sum \nolimits _{i=1}^{j} c_{i}\tag{19}\end{equation*}

$\lambda _{F}$ is the total degrees of freedom of machine movement space, $n_{L}$ is the total number of rods in the machine, $c_{i}$ is the degree of restraint on the $i^{\mathrm {th}}$ joint, and $j$ is the total number of machine joints. This machine comprised two rod-less pneumatic cylinders, a fabric-clamping platform, and the following degrees of freedom:

\begin{equation*} \mathrm {DOF}=6\times \left ({3-1 }\right)-\left ({2\times 5 }\right)=2\tag{20}\end{equation*}

Therefore, in terms of spatial movement, this machine had two degrees of freedom.

## IV. Experimental Results and Discussion

To verify the feasibility of the TDPSMSP, this study designed and then experimentally controlled 1) trajectory objectives and 2) fifth-order and hybrid polynomial trajectory-tracking models. This was done because data loss occurs when standard test signals, such as the step function, are used in pneumatic servo systems. Hybrid polynomial trajectory-tracking models comprise a fifth-order polynomial model and a sinusoidal function model. The designed fifth-order polynomial trajectory-tracking model has the following equation:

\begin{equation*} x_{d}\mathrm {(t)}=\sum \nolimits _{i=0}^{5} {a_{i}t^{i}}\tag{21}\end{equation*}

where

\begin{align*} a_{0}=&x_{d_{0}} \\ a_{1}=&\dot {x_{d_{0}}} \\ a_{2}=&\frac {1}{2}\ddot {x_{d_{0}}} \\ a_{3}=&\frac {1}{2t_{f}^{3}}[20x_{d_{f}}-20x_{d_{0}}-(8\dot {x_{d_{f}}}+12\dot {x_{d_{0}}})t_{f} \\&-\,3(\ddot {x_{d_{0}}}-\ddot {x_{d_{f}})}t_{f}^{2} \\ a_{4}=&\frac {1}{2t_{f}^{4}}[30x_{d_{0}}-30x_{d_{f}}+\left ({14\dot {x_{d_{f}}}+16\dot {x_{d_{0}}} }\right)t_{f} \\&+\,3(\ddot {x_{d_{0}}}-2\ddot {x_{d_{f}})}t_{f}^{2} \\ a_{5}=&\frac {1}{2t_{f}^{5}}[12x_{d_{f}}-12x_{d_{0}}-\left ({6\dot {x_{d_{f}}}+6\dot {x_{d_{0}}} }\right)t_{f}\mathrm {-(}\ddot {x_{d_{0}}}-\ddot {x_{d_{f}})}t_{f}^{2}\end{align*}

$x_{d_{0}}$, $\dot {x_{d_{0}}}$, and $\ddot {x_{d_{0}}}$ are the location, velocity, and acceleration of the TDPSMSP at time 0, respectively, and $x_{d_{f}},\dot {x_{d_{f}}}$, and $\ddot {x_{d_{f}}}$ are the location, velocity, and acceleration of the TDPSMSP at time $t_{f}$, respectively.

**FIGURE 4.**
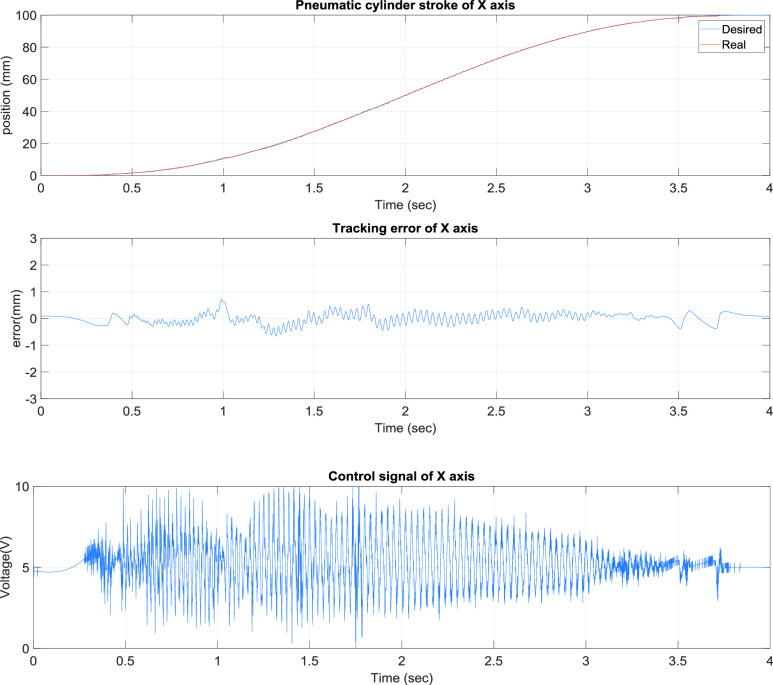
x-Axis tracking control results of the two-dimensional pneumatic servomechanism-based mask sewing platform fifth-order polynomial trajectory at 100 mm and a movement time of 4 s.

**FIGURE 5.**
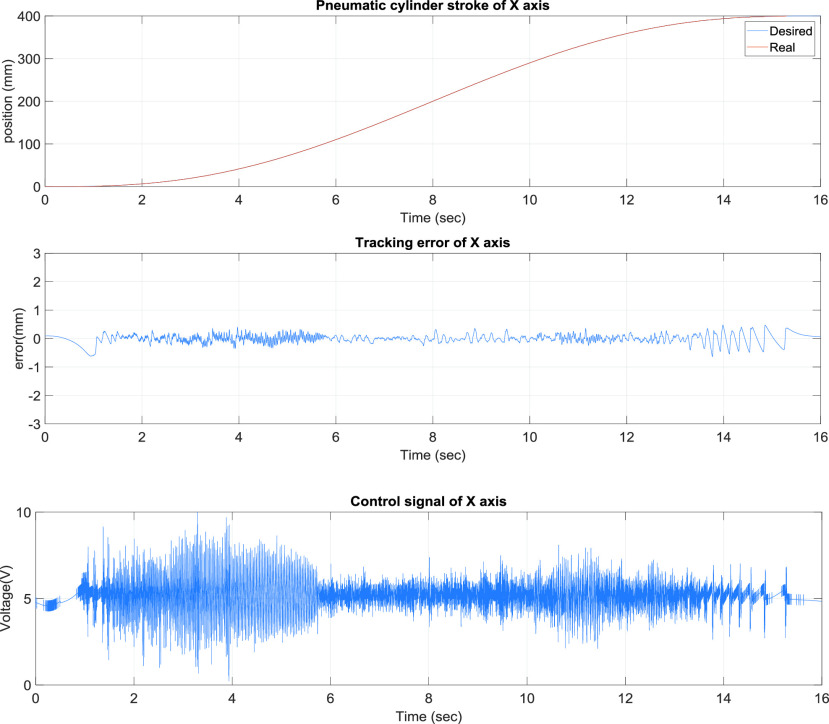
x-Axis tracking control results of the two-dimensional pneumatic servomechanism-based mask sewing platform fifth-order polynomial trajectory at 400 mm and a movement time of 16 s.

Figure 4 presents the x-axis tracking control results of the TDPSMSP fifth-order polynomial trajectory at 100 mm and a movement time of 4 s. Real-time trajectory-tracking control yielded errors within ±0.8 mm. The pneumatic servo valve was continually modified and compensated during movement to ensure favorable tracking performance. Figure 5 presents the x-axis tracking control results of the TDPSMSP fifth-order polynomial trajectory at 400 mm and a movement time of 16 s for a large stroke. Real-time trajectory-tracking control yielded errors within ±0.7 mm. The pneumatic servo valve was continually modified and compensated during movement to ensure favorable tracking performance. To investigate the performance of the rod-less pneumatic cylinder system in both forward and reverse directions (directions of movement required by the fabric-clamping platform), this study planned hybrid path models (sinusoidal trajectories with fifth-order polynomial trajectories) at $\mathrm {t}\le \mathrm {30~sec}$, fifth-order polynomial trajectories at 300 mm and 12 s, and sinusoidal trajectories at an amplitude of 100 mm and frequency of $0.2\pi $.

**FIGURE 6.**
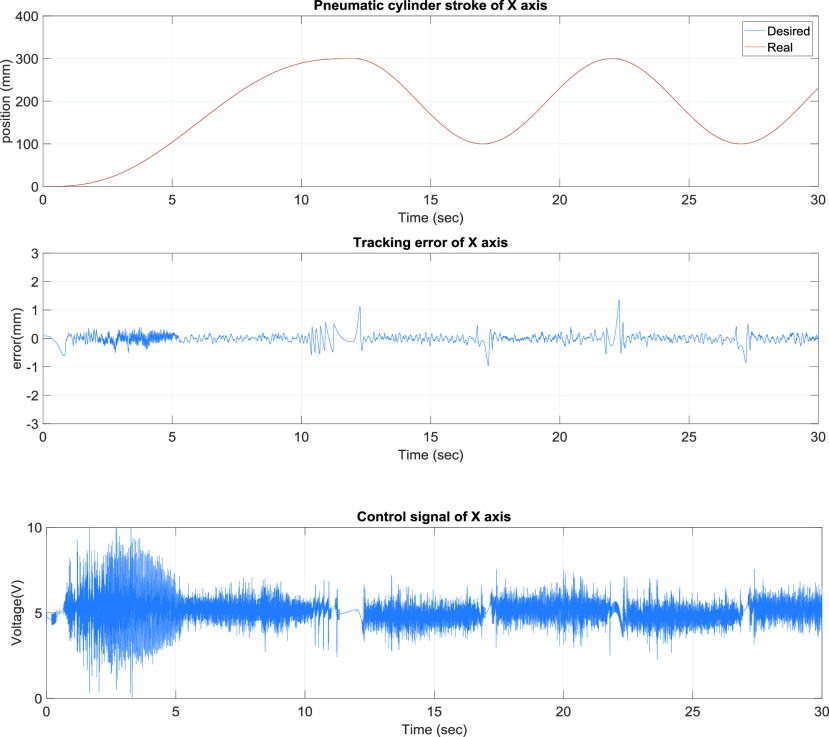
$x$-Axis control results of the two-dimensional pneumatic servomechanism-based mask sewing platform hybrid trajectories at $\mathrm {t}\le \mathrm {30~sec}$, fifth-order polynomial trajectories at 300 mm and 12 s, and sinusoidal trajectories at an amplitude of 100 mm and frequency of $0.2\pi $.

**FIGURE 7.**
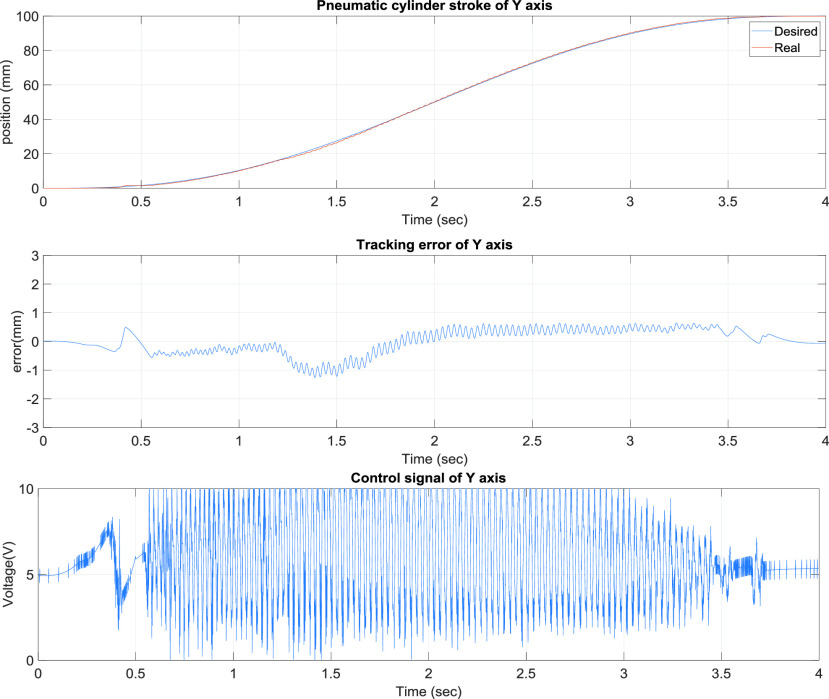
y-Axis tracking control results of the two-dimensional pneumatic servomechanism-based mask sewing platform fifth-order polynomial trajectory at 100 mm and a movement time of 4 s.

Figure 6 presents the x-axis control results of the TDPSMSP, which had relatively larger errors when the rod-less pneumatic cylinder alternated directions. Nevertheless, the pneumatic servo valve signals were continually modified and compensated during movement to ensure favorable system tracking results. Another axis, y-axis, is also controlled simultaneously. Figure 7 presents the y-axis tracking control results of the TDPSMSP fifth-order polynomial trajectory at 100 mm and a movement time of 4 s. Real-time trajectory-tracking control yielded errors within ±1.2 mm larger than x-axis’s errors because of the x-axis loading on the y-axis. The pneumatic servo valve was continually modified and compensated during movement to ensure favorable tracking performance under the x pneumatic cylinder loading.

**FIGURE 8.**
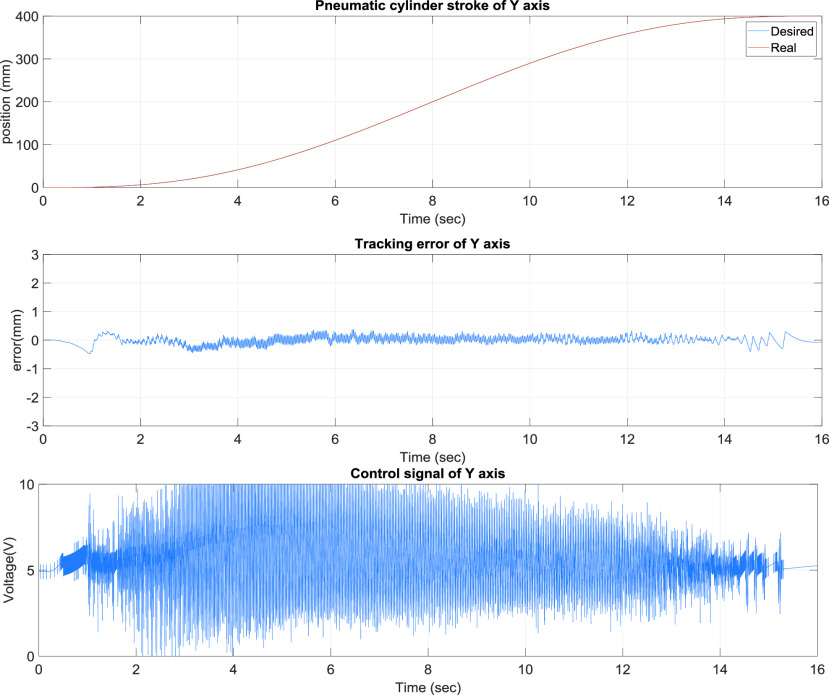
y-Axis tracking control results of the two-dimensional pneumatic servomechanism-based mask sewing platform fifth-order polynomial trajectory at 400 mm and a movement time of 16 s.

**FIGURE 9.**
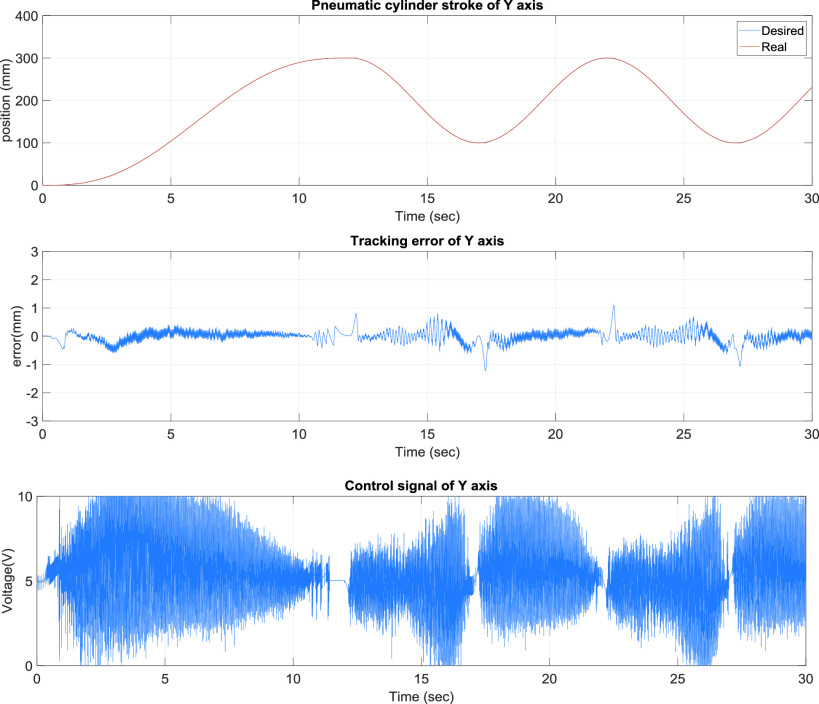
$y$-Axis control results of the two-dimensional pneumatic servomechanism-based mask sewing platform hybrid trajectories at $\mathrm {t}\le \mathrm {30~sec}$, fifth-order polynomial trajectories at 300 mm and 12 s, and sinusoidal trajectories at an amplitude of 100 mm and frequency of $0.2\pi $.

Figure 8 presents the y-axis tracking control results of the TDPSMSP fifth-order polynomial trajectory at 400 mm and a movement time of 16 s for a large stroke. Real-time trajectory-tracking control yielded errors within ±0.5 mm. The pneumatic servo valve was continually modified and compensated during movement to ensure favorable tracking performance. Figure 9 presents the y-axis control results of the TDPSMSP under hybrid trajectories at $\mathrm {t}\le \mathrm {30~sec}$, fifth-order polynomial trajectories at 300 mm and 12 s, and sinusoidal trajectories at an amplitude of 100 mm and frequency of $0.2\pi $. Relatively larger errors are ±1.2 mm, when the rod-less pneumatic cylinder alternated directions. Nevertheless, the pneumatic servo valve signals were continually modified and compensated during movement to ensure favorable system tracking results. As evident in Figures 4–9, changes in the control voltage can reduce errors in the trajectory-tracking results. However, the applied control voltage was not switched at the maximum values of +5 V and −5 V. Thus, the performance and lifetime of the pneumatic servo valve were within the requisite tolerance. Table 3 compares this study’s results, against those of [12]–[14], for the intelligent parameter adjustment feature with sliding-mode controller—for the S curve and polynomial trajectory. Relative to other controllers, this study’s controller performed best, with a root mean square error (RMSE) of 0.1563 mm for the real-time path tracking servo system.

**TABLE 3:** Comparisons of the Intelligent Parameter Adjustment With Sliding Mode Controller and Relevant Works for S Curve

Controller	RMSE (mm)
Ref. [12]	0.2640
Ref. [13]	0.2248
Ref. [14]	0.5873
Proposed method	0.1563

**FIGURE 10.**
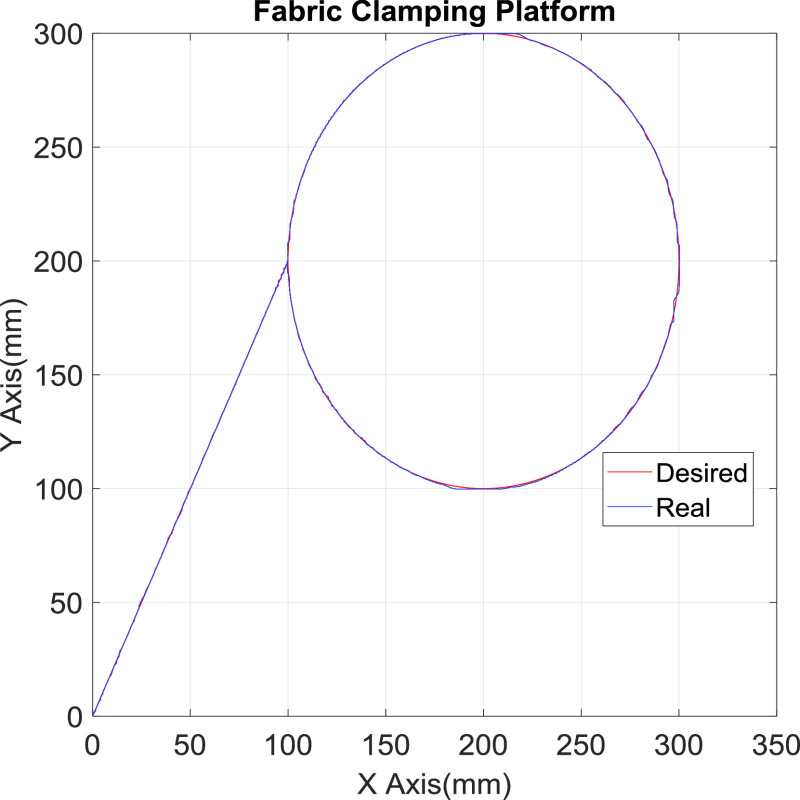
The circular tracking path results for the fabric clamping platform.

**FIGURE 11.**
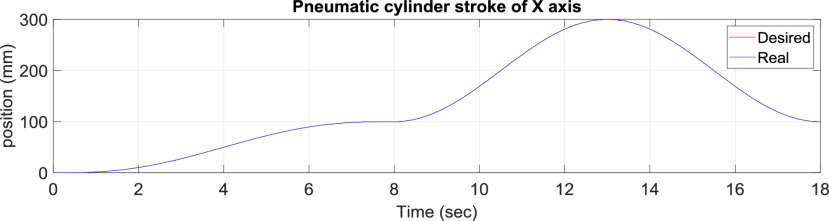
x-Axis pneumatic cylinder stroke results for the circular tracking path of the fabric clamping platform.

**FIGURE 12.**
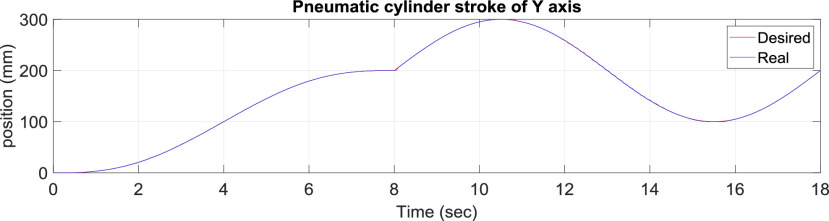
y-Axis pneumatic cylinder stroke results for the circular tracking path of the fabric clamping platform.

**FIGURE 13.**
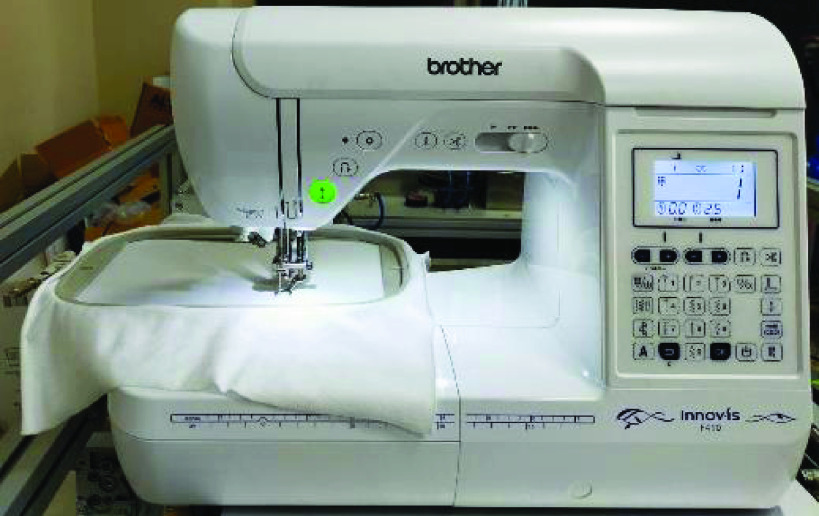
Layout of sewing process.

**FIGURE 14.**
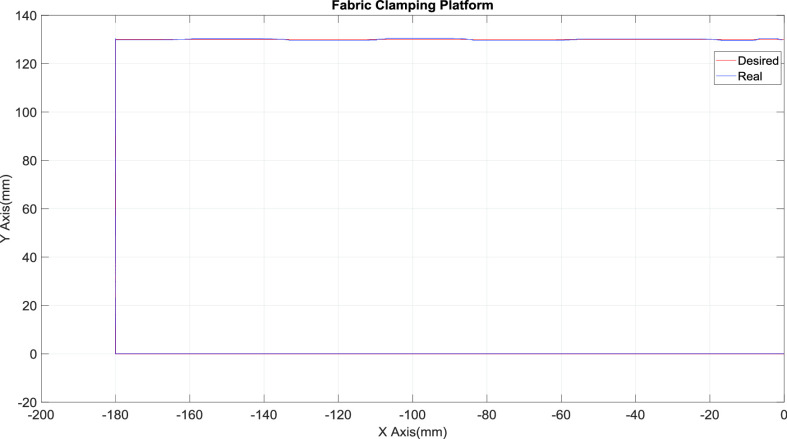
Tracking path results of the fabric-clamping platform.

**FIGURE 15.**
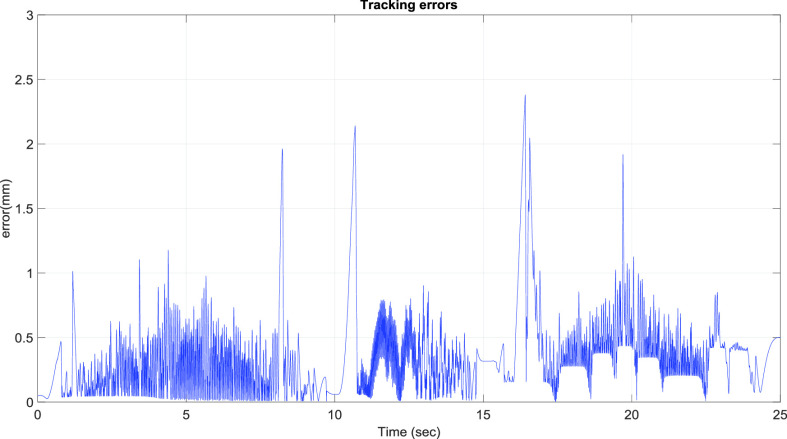
Tracking path errors of the fabric-clamping platform.

Figure 10 presents the results for the circular tracking path of the fabric-clamping platform. According to the results, the fabric-clamping platform moved from (X, Y) = (0 mm, 0 mm) to (X, Y) = (100 mm, 200 mm) in 8 s along fifth-order polynomial trajectories for each axis. As for circular trajectories, the platform required 10 s to cover one revolution of a 200-mm diameter trajectory. Figure 11 and Figure 12 present the results for each pneumatic cylinder stroke of the fabric-clamping platform when a circular tracking path was taken. The sampling frequency was 1000 Hz in the experiments. The sewing process is shown in Figure 13. The fabric-clamping platform covered the fabric by the pneumatic real-time servo control system for fabric moving and the sewing machine was for needle sewing. The pneumatic platform and the sewing machine should cooperate precisely. Otherwise, the needle will be broken. Figure 14 shows tracking path results of the fabric clamping platform for mask production. The red line is desired path for the fabric clamping platform moving and the blue line is experimental results. The fabric clamping platform moves from (X, Y) = (0mm, 0mm) to (X, Y) = (−180mm, 0mm) in 10 seconds by fifth-order polynomial trajectories and then moves from (X, Y) = (−180mm, 0mm) to (X, Y) = (−180mm, 130mm) in 5 seconds by fifth-order polynomial trajectories. Finally, the fabric clamping platform moves from (X, Y) = (−180mm, 130mm) to (X, Y) = (0mm, 130mm) in 10 seconds by fifth-order polynomial trajectories. Figure 15 presents the tracking path errors of the fabric-clamping platform. The error results indicated tracking errors that increased suddenly to >2 mm in acceleration and deceleration.

**FIGURE 16.**
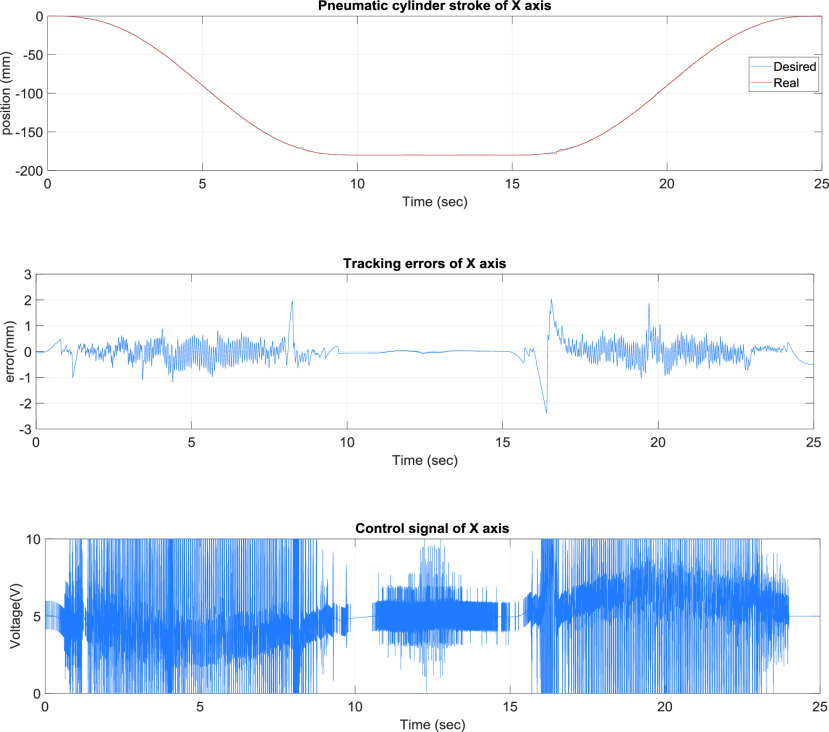
Mask-production tracking control results for the x axis.

**FIGURE 17.**
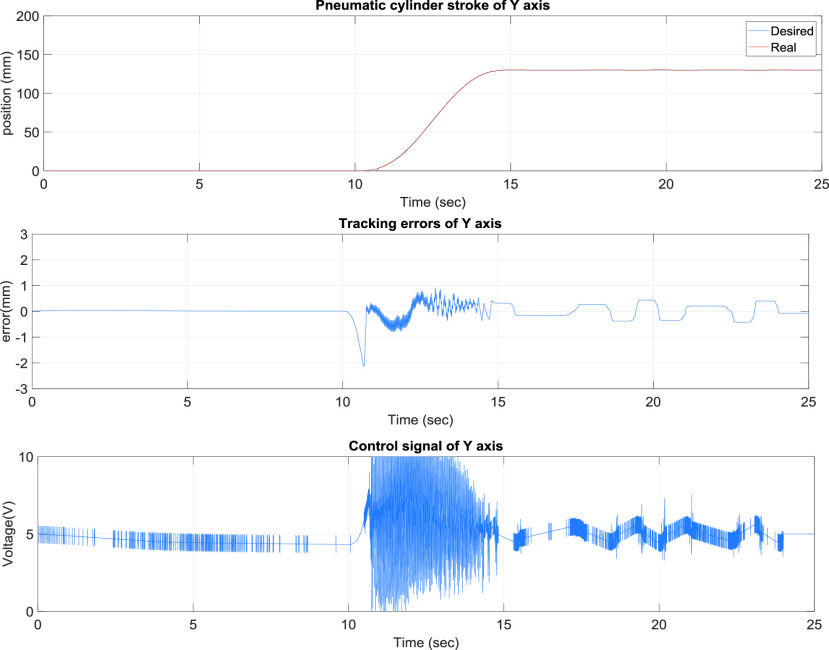
Mask-production tracking control results for the y axis.

**FIGURE 18.**
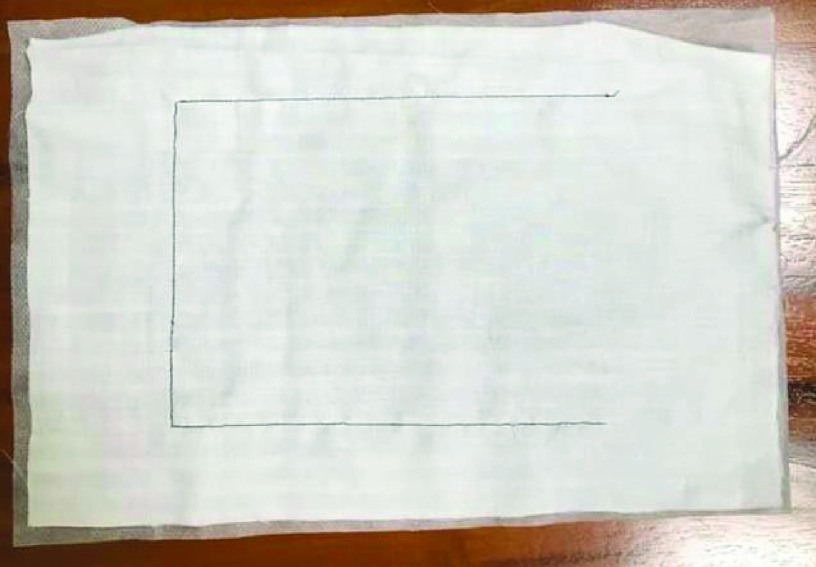
The tracking path results for the mask production.

**FIGURE 19.**
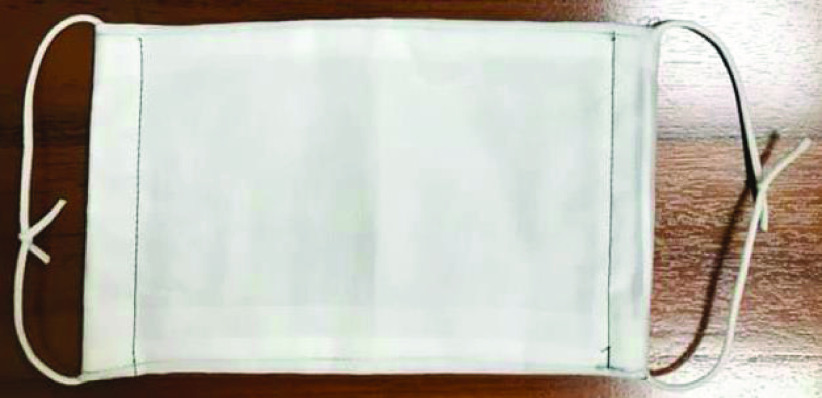
The finished mask product.

However, the overall error could be controlled to levels that are acceptable for mask production. Errors larger than those noted in this study will result in 1) breakage of the needle and thread and 2) a mismatch between the sewing machine and pneumatic servo system. Smaller tracking errors result in a smaller sewing pitch. Thus, the tracking error was set to 2 mm (< 5 mm) in this study. Figure 16 and Figure 17 illustrate the system responses, tracking errors, and control signals for each actuator during mask production for the x and y axes, respectively. The tracking errors could be controlled to be within 2.3 mm and 2.05 mm for x and y axis, respectively; these errors resulted from the friction forces during acceleration and deceleration. Figure 18 shows the tracking paths results of the mask production. Figure 19 shows the finished mask product produced by the TDPSMSP automatically. The root mean square error (RMSE) of the final mask product is 0.495 mm. Table 4 compares the hot press forming method with the sewing method, as used in mask production.

**TABLE 4:** Comparisons Between Hot Press Forming Method and Sewing Method in Mask Production

	HOT PRESS FORMING METHOD	SEWING METHOD
Material	PP fabric and composite fiber	Cotton, double gauze, PP fabric
Product diversification	Single	Diverse, customized

## V. Conclusion

This study formulated a novel TDPSMSP that can perform real-time trajectory tracking and control under a real-time MATLAB Simulink environment through an intelligent parameter adjustment feature with a sliding-mode controller. The rod-less pneumatic servo system was experimentally shown to provide impressive trajectory-tracking precision on both fifth-order polynomial and hybrid trajectories. Furthermore, mask production was successfully implemented in the experimental test rig. This study’s controller performed best relative to its counterparts; it had RMSEs of 0.1563 and 0.495 mm for the real-time path tracking servo system and the final mask product, respectively. By adopting the TDPSMSP in automated manufacturing and in sewing-related operations, mask manufacturers can substantially reduce labor costs as well as increase productivity, yield, and efficiency. Furthermore, the proposed novel TDPSMSP can be applied in conjunction with different sewing machines for mask production at various speeds. Pneumatic cylinder speeds of >30 mm/s will result in incomplete sewing results; this is because the upper and lower needle cannot match the cotton threads to be used during sewing. Conversely, pneumatic cylinder speeds of < 15 mm/s result in unacceptable errors in the final product. In the mask production experiment, the feed speed of fabric could reach 26 mm/s, which was greater than the 14.2 mm/s sewing speed of the sewing machine used.
